# Why intensivists want chest radiographs

**DOI:** 10.1186/s13054-015-0816-x

**Published:** 2015-03-05

**Authors:** Martijn Tolsma, Peter HJ van der Voort, Nardo JM van der Meer

**Affiliations:** Department of Anesthesiology & Intensive Care, Isala Clinics, Dokter van Heesweg 2, Zwolle, 8025 AB The Netherlands; Department of Intensive Care, Onze Lieve Vrouwe Hospital, Oosterpark 9, Amsterdam, 1091 AC The Netherlands; Department of Healthcare, Tias Nimbas Business School, Tilburg University, Warandelaan 2, Tilburg, 5037 AB The Netherlands; Department of Anesthesiology & Intensive Care, Amphia Hospital, Molengracht 21, Breda, 4818 CK The Netherlands

We would like to contribute to the ongoing discussion regarding different chest radiograph (CXR) strategies in the intensive care unit (ICU). In their review and meta-analysis in a recent issue of *Critical Care*, Ganapathy and colleagues [1] concluded that they found no harm associated with a restricted CXR strategy for ICU patients. On the other hand, they stated that the safety of abandoning routine CXRs for ICU patients was still uncertain.

Several investigators, including one large multicenter randomized trial [2], confirmed that performing an on-demand CXR strategy instead of a daily routine CXR strategy decreased the total number of CXRs significantly and was accompanied by an increase in their diagnostic efficacy but without any increase in adverse events or the use of other imaging studies. This leads to the question of what the exact indications for on-demand CXRs in critically ill patients are. And might there be certain patient groups that may still benefit from routine CXRs?

Another interesting point of view is the impact of an on-demand CXR strategy on workflow and efficiency [3], where a number of issues still need to be addressed. For example, can certain ICU patients safely be transferred to the ward without a CXR before? What is the impact of ‘negative’ CXR findings on this workflow and on our personal clinical decision-making? And is it possibly more (cost) efficient for a radiology department to perform multiple routine CXRs during a morning round instead of performing several single CXRs during the day and at night?

Our recent web study among Dutch intensivists showed that, nowadays, in line with the current evidence, a daily routine CXR strategy is used significantly less frequently than one decade ago [4]. However, surrogate routine strategies like performing a routine CXR on certain fixed days a week or only on the first days of admission have become more popular. Intensivists still assume the value of these CXRs to be higher than the efficacy that is reported in the literature and this might be due to the clinical value of negative findings, which has not been studied before. And most clinicians, including surgeons and consulting physicians, probably are used to the performance of CXRs for their ICU patients.

A lot of ICUs seem to have no clear protocol regarding specific indications for CXRs, and the least experienced ICU clinicians may request more CXRs. The importance of (negative) CXR findings on workflow, efficiency, and clinical decision-making may be larger than is estimated. To further reduce the number of unnecessary CXRs safely, we recommend that ICU departments design a local protocol regarding their CXR indications. In addition, ideally, experienced intensivists should request these CXRs for the mentioned reasons.

## Abbreviations

CXR: Chest radiograph
ICU: Intensive care unit
